# Changes in the diversity and composition of gut microbiota in pigeon squabs infected with *Trichomonas gallinae*

**DOI:** 10.1038/s41598-020-76821-9

**Published:** 2020-11-17

**Authors:** Feng Ji, Dongyan Zhang, Yuxin Shao, Xiaohan Yu, Xiaoyong Liu, Dacong Shan, Zheng Wang

**Affiliations:** Institute of Animal Husbandry and Veterinary Medicine, Beijing Academy of Agriculture and Forestry Sciences, Beijing, China

**Keywords:** Microbiology, Systems biology

## Abstract

Pigeons, as the only altricial birds in poultry, are the primary *Trichomonas gallinae* (*T. gallinae*) host. To study the effects of *T. gallinae* infection on gut microbiota, we compared the microbiota diversity and composition in gastrointestinal (GI) tracts of pigeons at the age of 14 and 21 day with different degrees of *T. gallinae* infection. Thirty-six nestling pigeons were divided into three groups: the healthy group, low-grade and high-grade trichomonosis group. Then, the crop, small intestine and rectum contents were obtained for sequencing of the 16S rRNA gene V3–V4 hypervariable region. The results showed that the microbiota diversity was higher in crop than in small intestine and rectum, and the abundance of *Lactobacillus* genus was dominant in small intestine and rectum of healthy pigeons at 21 days. *T. gallinae* infection decreased the microbiota richness in crop at 14 days. The abundance of the *Firmicutes* phylum and *Lactobacillus* genus in small intestine of birds at 21 days were decreased by infection, however the abundances of *Proteobacteria* phylum and *Enterococcus, Atopobium, Roseburia, Aeriscardovia* and *Peptostreptococcus* genus increased. The above results indicated that crop had the highest microbiota diversity among GI tract of pigeons, and the gut microbiota diversity and composition of pigeon squabs were altered by *T. gallinae* infection.

## Introduction

The pigeon is the fourth largest poultry product in China, and there’re approximately 40 million pairs of breeding pigeons in our country. More than 600 million commercial pigeons are sold annually. Pigeon meat is consumed as a kind of nutritional food, and is gaining popularity among consumers in Europe mainly France and Italy^1,2^, United States^3^, Pakistan^4^ and Japan^5^. Additionally, pigeon is also popular as racing and fancy bird in many areas of the world.

*Trichomonas gallinae* (*T. gallinae*) is the causative agent of canker, leading to serious losses and high mortality, especially in young birds^6^. The rock pigeon (*Columba livia*) is the primary host of *T. gallinae* and has been considered responsible for the worldwide distribution of this protozoal infection^7,8^. In China, several reports have revealed a high prevalence in the provinces of Sichuan, Guangdong and Shandong^9,10^. Trichomoniasis has also been identified in a wide range of noncolumbiform species, including turkeys, chickens, falcons, hawks and songbirds^11–14^. Most recently, populations of passerine birds in Great Britain, Fennoscandia, France, Germany, Slovenia, and Canada have been reported to suffer from *T. gallinae* epidemics^15–20^.

It was reported that the infection rate of nestling pigeons was higher than that of adolescent and breeding pigeons^21^. Young pigeons, as altricial birds, are unable to take the food voluntarily until 4 weeks old, and squabs are fed by the parents in a mouth-to-mouth way. It has been suggested that the transmission of parasites via crop milk from infected parent birds to squabs seems most efficient for establishing an infection^7^. However, little information is available concerning the effects of trichomoniasis infection on the composition, structure and dynamics of microbial populations residing in gastrointestinal (GI) tract of birds. Only Taylor et al.^22^ reported that the oral microbiota of Cooper’s hawks differed between nestlings and older birds, and speculated oral microbial community composition may correlate with the age-specific differences in susceptibility to *T. gallinae*. We hypothesized that *T. gallinae* infection might influence the microbial diversity and composition from upper GI tract of pigeons at earlier days of age. Therefore, in our present study, we performed high-throughput sequencing to compare the diversity and composition of the microbiota from different segments of digestive tract in healthy and *T. gallinae* infected pigeon squabs at different age. This study provided us with fundamental knowledge that will facilitate the development of a new concept, probiotics, to treat *T. gallinae* infection in pigeon production in the future.

## Results

A total of 237 squabs were diagnosed to obtain the experimental birds in this study. Our previous study has shown that the infection rate of *T. gallinae* was 77.8% in 689 pigeon squabs, whereas the prevalence was only 30.0% in 393 parent pigeons in our district (data not published). Among the infected squabs, the proportions of low-grade (LG) and high-grade (HG) *T. gallinae* infected birds were similar, which were around 30%. The birds with similar body weight (336.94 ± 43.75 g and 446.78 ± 38.01 g at the age of 14-day and 21-day, respectively) and without any clinical symptoms were used in further study.

### DNA sequence data from all treatments

After chimeras were filtered out and low-quality sequences were removed, 5,641,792 valid sequences were obtained in total from 108 samples, and the sequence number of 31,139 per sample after normalization was used for further alpha and beta diversity analyses. The filtered high-quality sequences were assigned to 800 OTUs at 97% similarity, with an average of 445.57 bp.

### Alpha diversity of the gut microbiota of control pigeon squabs

The values of ACE (*Q* = 0.049) and Chao1 (*Q* = 0.091) for rectal samples of 14-day-old squabs were significantly lower than those of 21-day-old squabs, indicating that the community richness in this segment increased with age. However, there was no significant difference between groups of 14 days and 21 days in other indexes (data not shown). The indexes in this study showed an increasing tendency in community richness and diversity of the GI microbiota with increasing age over the experimental period; however, statistical analyses indicated that the species richness and diversity were not significantly affected by age in crop and small intestine.

**Figure 1:**
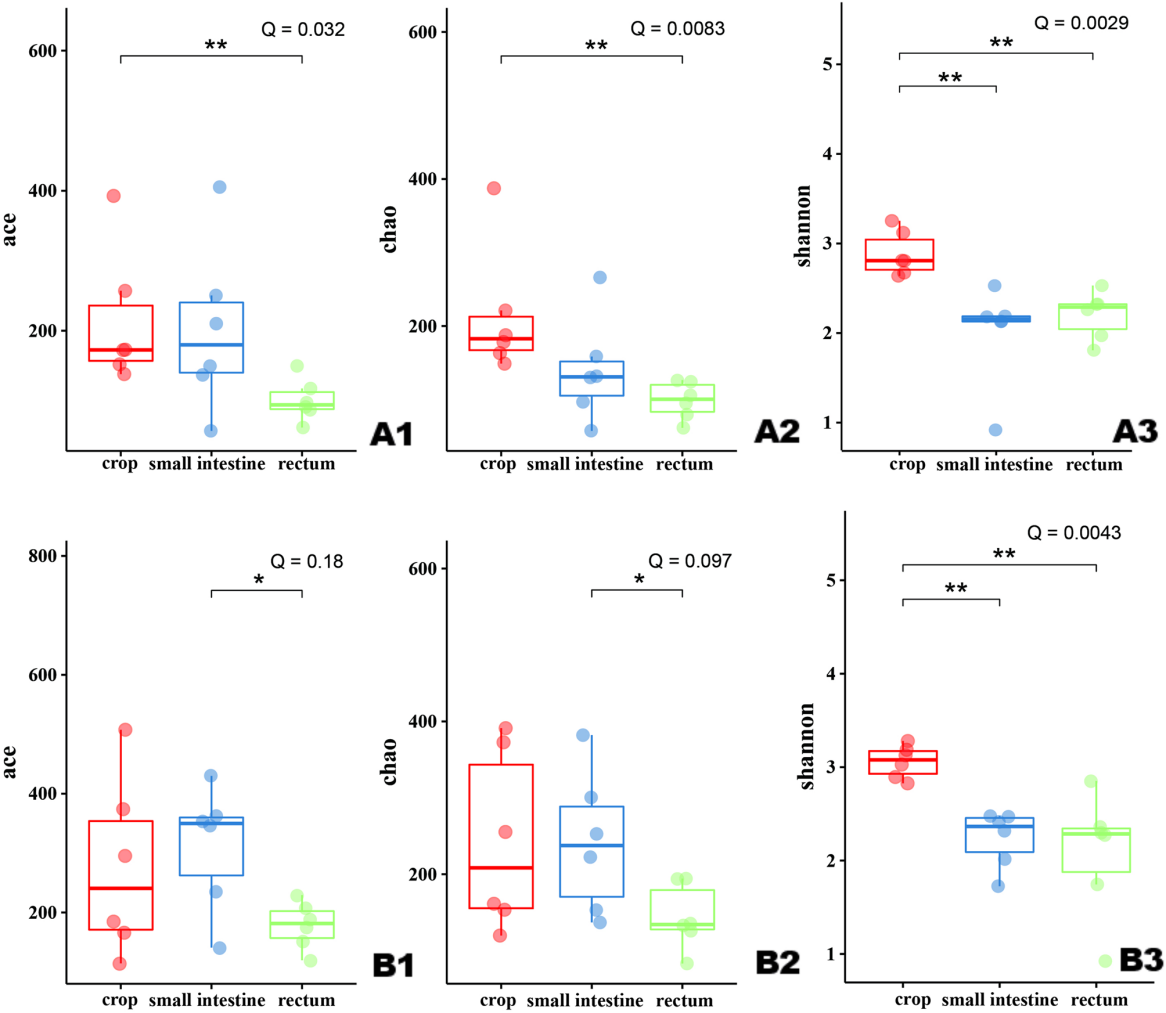
Microbial alpha diversity indexes of the samples (n = 18) from crop, small intestine and rectum of control squabs, the data are presented as median with IQR; (**A**) 14-day-old; (**B**) 21-day-old.

The microbial diversity indexes of the samples from different GI segments of control squabs are presented in Fig. 1. The species diversity was significantly decreased from the upper GI segment (crop) to the hindgut (small intestine and rectum) according to the Shannon index (*Q* < 0.01, Fig. 1A3,B3). There was no significant difference in the diversity index between the small intestine and rectum of squabs. A significant change toward lower microbial abundance in the hindgut in relation to that in the crop was also observed at 14 days of age (*Q* < 0.05, Fig. 1A1,A2). However, the change was not significant at 21 days old birds, indicating the microbial abundance increased in the hindgut, especially small intestine, with age (Fig. 1B1,B2).

### Relationships among the microbiota of different GI segments in control pigeon squabs

**Figure 2:**
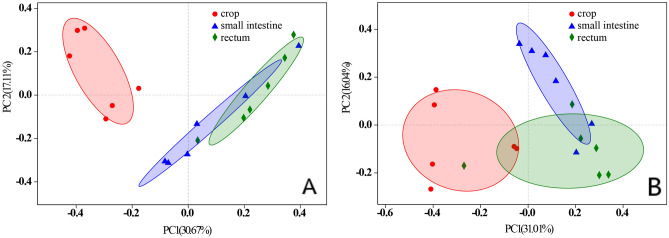
Multiple sample PCoA analysis (n = 18) among the GI segments of control squabs; (**A**) 14-day-old; (**B**) 21-day-old.

Principal coordinate analysis (PCoA) was conducted to identify the similarities and differences among the microbiota of three GI segments (Fig. 2). Comparison of the microbiota from the different GI segments showed that the taxonomic composition of the crop was totally separate from those of the small intestine and rectum at the age of 14 days (Fig. 2A), and the difference became less at 21 days old (Fig. 2B). The microbiota communities of the small intestine and rectum overlapped a little, indicating that the community structures of the two segments were more similar in the study.

**Figure 3:**
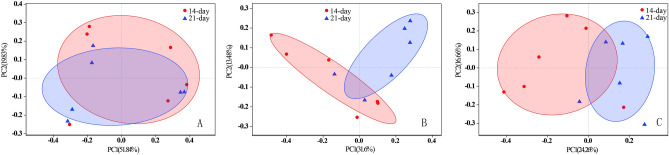
Multiple sample PCoA analysis (n = 12) between the two ages of control squabs; (**A**) crop; (**B**) small intestine; (**C**) rectum.

Comparison of the microbiota indicated that there was no difference between the taxonomic compositions of crop samples from different ages, while the situations were different in small intestine and rectum of the two ages, respectively (Fig. 3). It was speculated that the characterized microbiota gradually dominated in the hind gut as aging.

### Shifts in the microbiota of control pigeon squabs of different ages and GI segments

*Firmicutes*, *Proteobacteria*, *Bacteroidetes, Actinobacteria*, and *Tenericutes* were the dominant phyla in the GI tract of pigeon squabs (Supplemental Fig. 1), representing more than 96% of taxa in the crop, small intestine, and rectum, respectively. In the present study, we found that some taxa abundances changed among different GI segments. For example, *Firmicutes* was more abundant in the small intestine and rectum than in the crop of squabs at the age of 14 days and 21 days (83.96% in small intestine, 86.38% in rectum, and 23.07% in crop, *Q* = 0.04629, 14 days old, Supplemental Fig. 1A; 96.01% in small intestine, 91.11% in rectum, and 21.99% in crop, *Q* = 0.01011, 21 days old, Supplemental Fig. 1B). Oppositely, the abundance of *Proteobacteria* decreased significantly in the small intestine and rectum in relation to that in the crop. The relative abundances of *Bacteroidetes*, *Tenericutes*, and *Fusobacteria* found in the crop were the highest among the three GI segments of pigeon (*Q* < 0.05, Supplemental Fig. 1A2,B2). The proportion of *Gracilibacteria* in the crop was also the highest among the GI segments (*Q* < 0.1, Supplemental Fig. 1A2,B2).

However, no significant effects of age were observed on the phyla proportions in crop, small intestine and rectum (*Q* > 0.1, data not shown).

**Figure 4:**
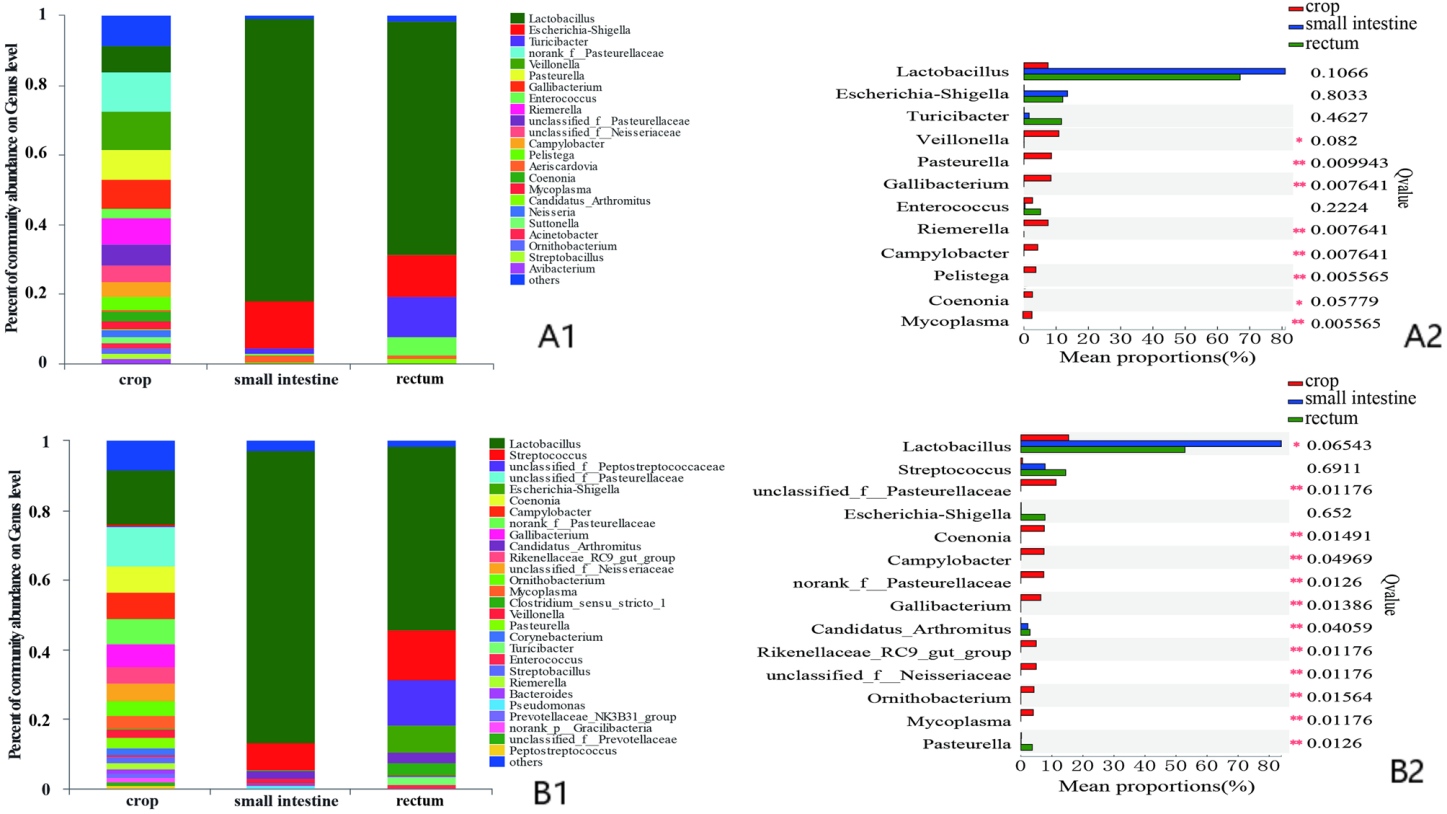
Changes in the relative abundances of the main bacterial communities (n = 12) on Genus level in the GI segments of control squabs; (**A1**) community bar plot at 14 days; (**A2**) Kruskal–Wallis H test bar plot at 14 days; (**B1**) community bar plot at 21 days; (**B2**) Kruskal–Wallis H test bar plot at 21 days.

There were significantly higher abundances of eight genera detected in the crop compared to the small intestine and rectum, including *Campylobacter* (*Q* = 0.0076), *Pasteurella* (*Q* = 0.0099), *Gallibacterium* (*Q* = 0.0076), *Riemerella* (*Q* = 0.0076), *Pelistega* (*Q* = 0.0056) and *Mycoplasma* (*Q* = 0.0056), *Veillonella* (*Q* = 0.082) and *Coenonia* (*Q* = 0.058), at the age of 14 days (Fig. 4A1,A2). However, the abundance of *Lactobacillus* was higher in the small intestine and rectum than in the crop (Fig. 4B1), and the difference was significant at the age of 21 days (*Q* = 0.065, Fig. 4B2). At this age, the proportions of *Campylobacter* (*Q* = 0.050), *Coenonia* (*Q* = 0.015), *Gallibacterium* (*Q* = 0.014), *Ornithobacterium* (*Q* = 0.016), and *Mycoplasma* (*Q* = 0.012) were higher in the crop than in the other GI segments in this study (Fig. 4B1,B2).

### Alpha diversity of the GI microbiota of pigeon squabs infected by *T. gallinae*

**Figure 5:**
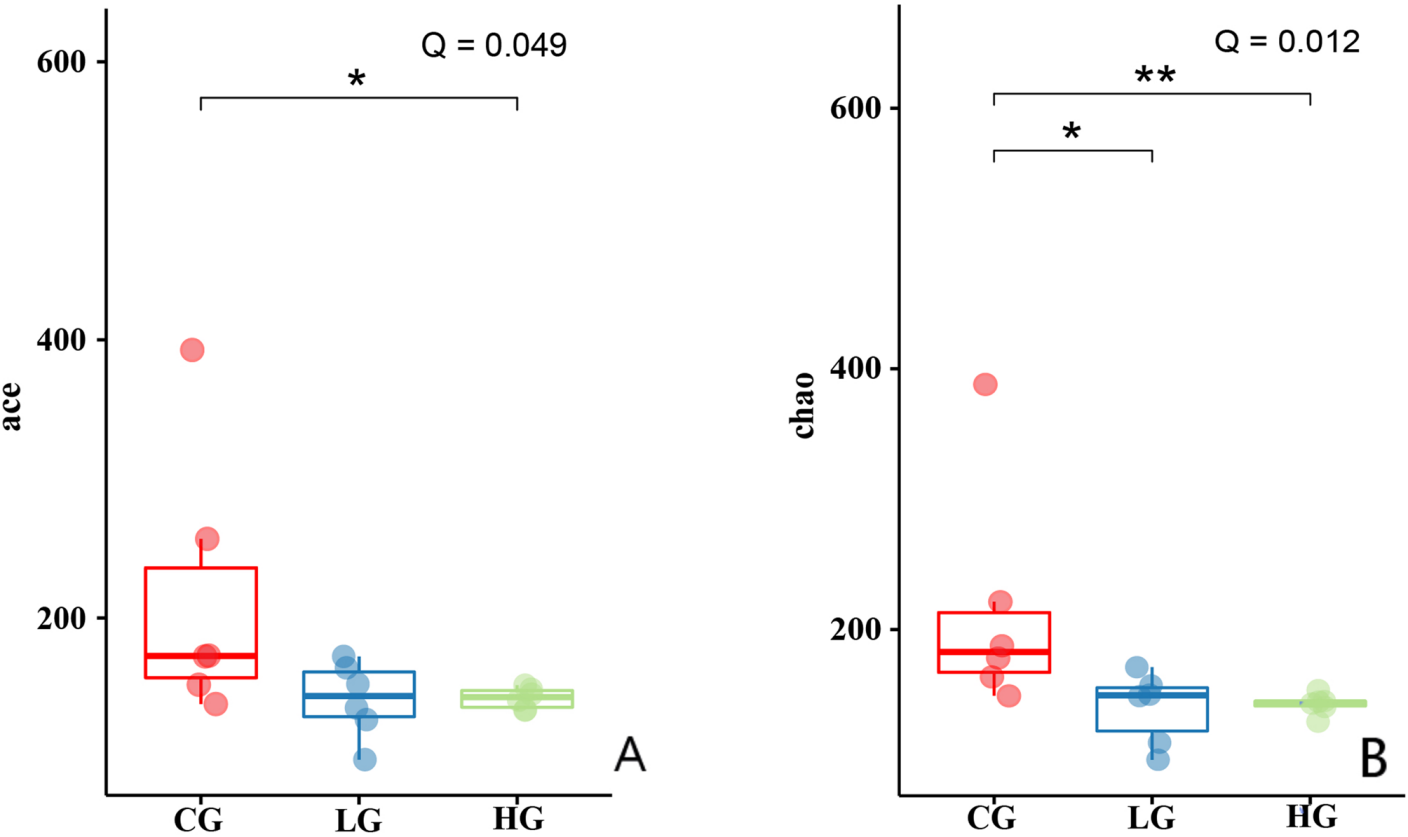
Effects of trichomonosis on estimated microbial richness of crop samples (n = 18) from14-day old squabs, the data are presented as median with IQR; (**A**) ACE index; (**B**) Chao 1 index.

**Figure 6:**
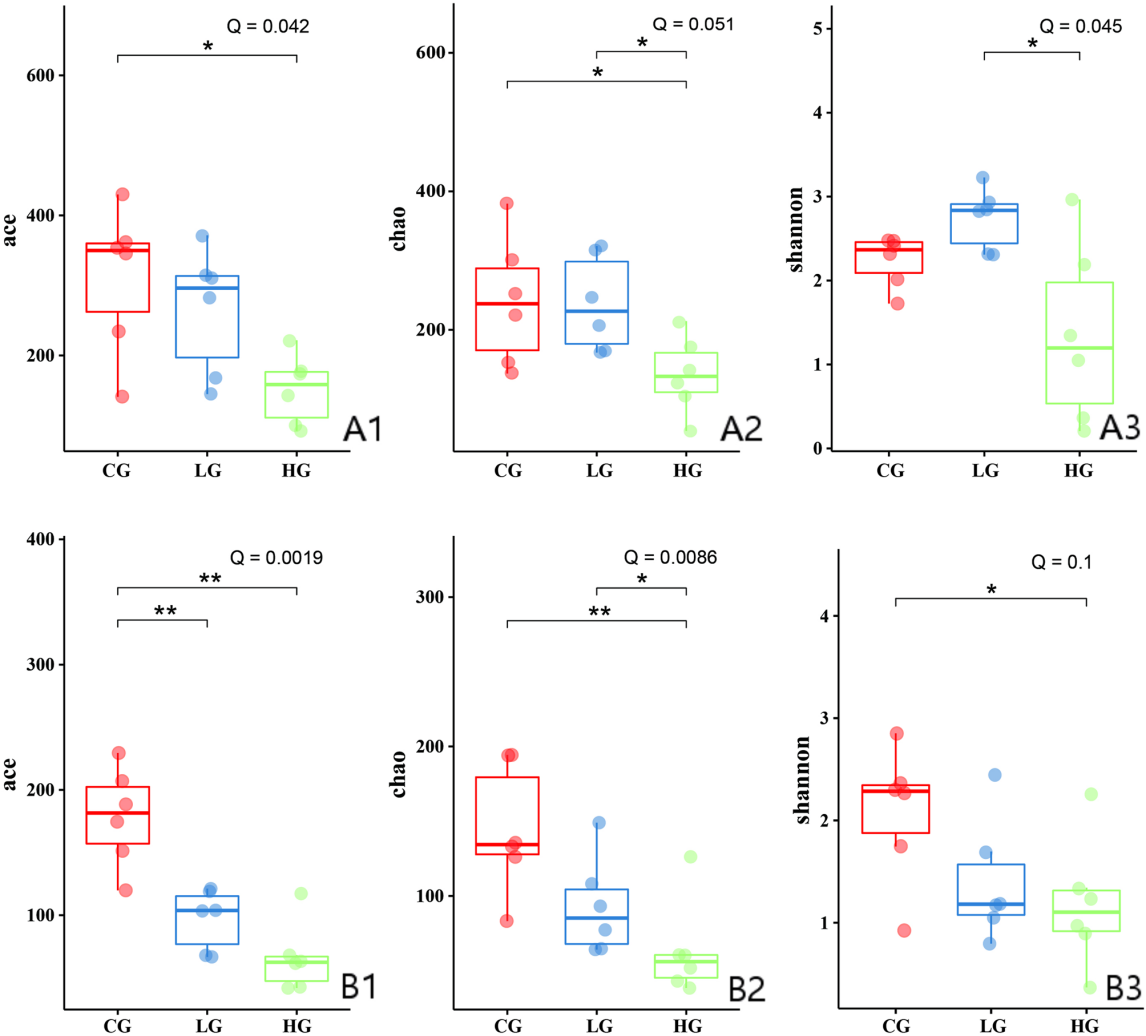
Effects of trichomonosis on microbial alpha diversity indexes of the samples (n = 18) from 21-day old squabs, the data are presented as median with IQR; (**A**) small intestine; (**B**) rectum.

The microbial diversity indexes of the samples from pigeon squabs infected by *T. gallinae* at the age of 14 and 21 days are shown in Figs. 5 and 6, respectively. At the age of 14 days, the ACE value of crop samples in the control group was significantly higher (*Q* < 0.05) than that in HG group, and the Chao1 of the control group was also significantly higher (*Q* < 0.05) than that in LG group. The results indicated the parasite infection decreased microbial abundance in the crop. However, statistical results indicated that the species richness and diversity in the small intestine and rectum were not affected by trichomonosis (data not shown).

At the age of 21 days, the alpha diversity was significantly decreased in small intestine and rectum of HG group compared with that in these GI segments of healthy birds (control group, CG), according to ACE and Chao1 values (*Q* < 0.05, Fig. 6A1,B1,B2). The differences in the abundance indexes between LG and HG group were significant for the Chao1 values of small intestinal and rectal contents, respectively (*Q* ≤ 0.05, Fig. 6A2,B2). The species diversity was significantly affected by severe trichomonosis in rectum (*Q* = 0.1, Fig. 6B3), and there was significant difference between LG and HG group in small intestine according to the Shannon index (*Q* < 0.05, Fig. 6A3).

### Effects of trichomonosis on the GI microbiota composition of pigeons

**Figure 7:**
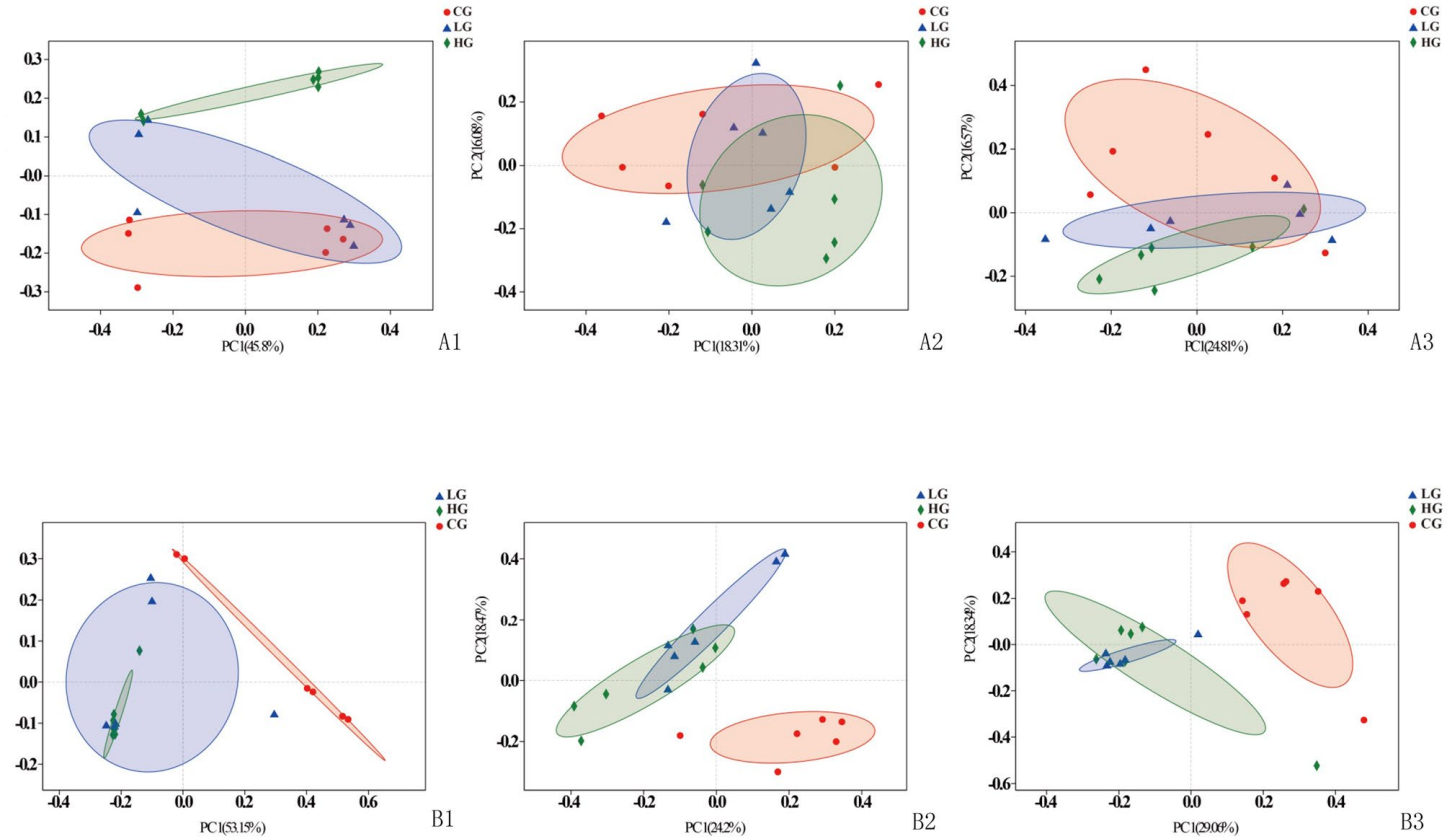
Multiple sample PCoA analysis (n = 18) among the CG, LG and HG infection groups at the age of 14 and 21 days, respectively: (**A1**) crop; (**A2**) small intestine; (**A3**) rectum of 14-day; (**B1**) crop; (**B2**) small intestine; (**B3**) rectum of 21-day.

Principal coordinate analysis (PCoA) was conducted to identify the similarities and differences in the microbiota of the samples among the groups of CG, LG and HG at the age of 14 and 21 days, respectively (Fig. 7). Comparison of the microbiota of the three groups showed that the taxonomic composition of the crop samples from HG birds was totally separated from the data of control birds at the age of 14 days, while the composition of the crop samples from LG and CG group were more similar (Fig. 7A1). The microbiota in small intestine and rectum among the three groups were not separated distinctly (Fig. 7A2,A3). The microbiota of CG group was quite different from those of LG and HG birds in not only crop but also the other GI segments at 21 days old (Fig. 7B1,B2,B3). The data confirmed that the effect of trichomonosis on the taxonomic composition appeared with increasing age, because the characterized microbial community gradually existed at the age of 21 days in this study. The taxonomic composition of samples from LG group was similar to that of samples from HG group (Fig. 7), indicating that the community structures of the two groups were similar in the study.

**Figure 8:**
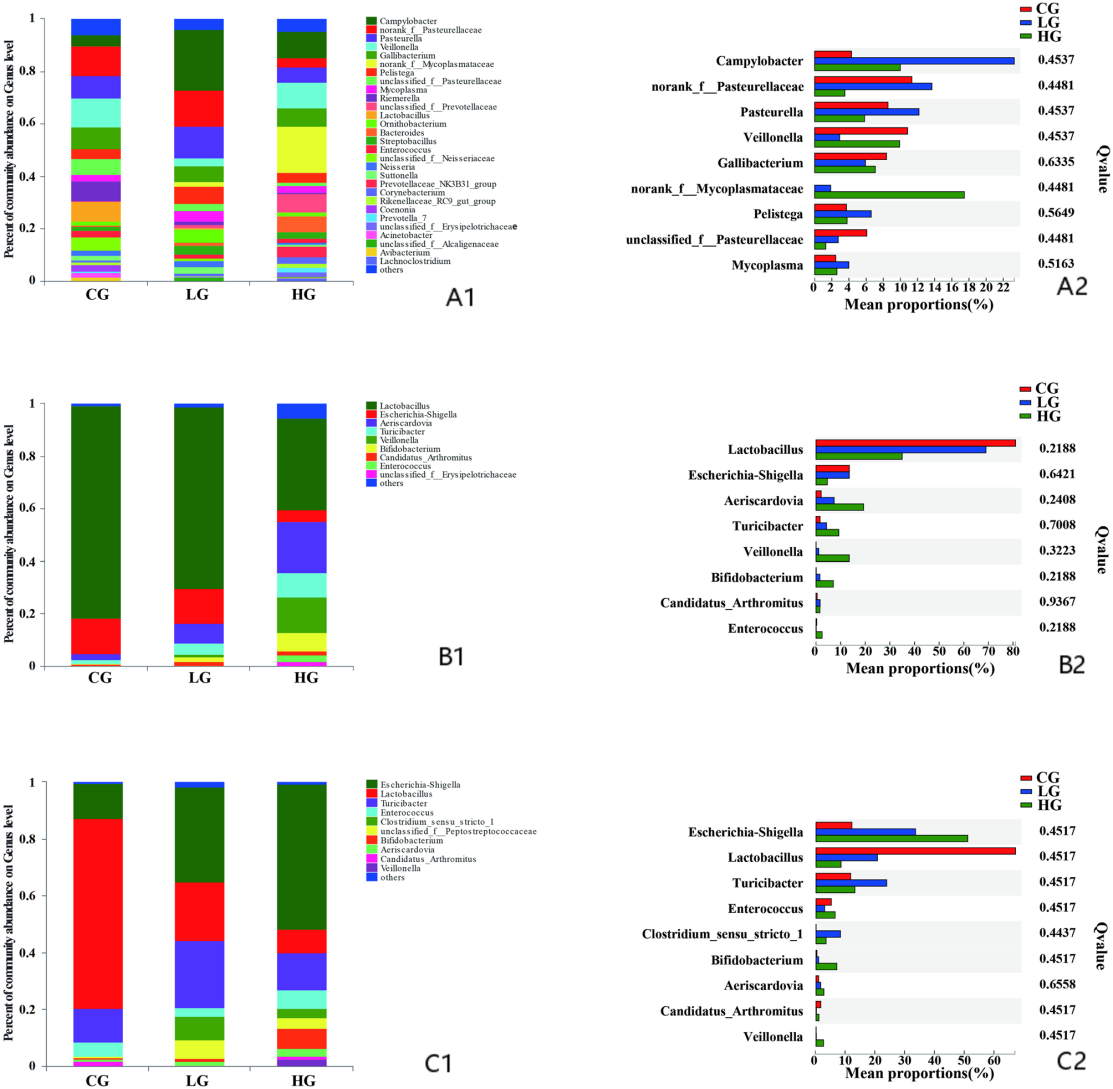
Changes in the relative abundances of the main bacterial communities (n = 18) on Genus level among the CG, LG and HG infection groups at the age of 14 days; (**A1**) community bar plot of crop samples; (**A2**) Kruskal–Wallis H test bar plot of crop samples; (**B1**) community bar plot of small intestinal samples; (**B2**) Kruskal–Wallis H test bar plot of small intestinal samples; (**C1**) community bar plot of rectal samples; (**C2**) Kruskal–Wallis H test bar plot of rectal samples.

At day 14, the composition of microbiota was not significantly affected on the phylum level in the crop, small intestine and rectum of birds with trichomonosis compared with control birds, except *Actinobacteria* increased (*Q* = 0.056) in the small intestine of infected birds (data not shown). At the genus level, the relative abundance of *Riemerella* was decreased in the crop of birds with severe trichomonosis (Fig. 8A1). The relative abundance of *Lactobacillus* was decreased in the small intestine and rectum of birds with trichomonosis (Fig. 8B1,C1). The percentages of *Aeriscardovia*, *Bifidobacterium*, and *Enterococcus* were increased in the small intestine of birds with trichomonosis (Fig. 8B1), and *Escherichia–Shigella* increased in the rectum of birds with infection (Fig. 8C1). However, there was no significant effect of trichomonosis on any microbiota proportions on Genus level in crop, small intestine and rectum according to the corrected *P*-values in this study (Fig. 8A2,B2,C2).

**Figure 9:**
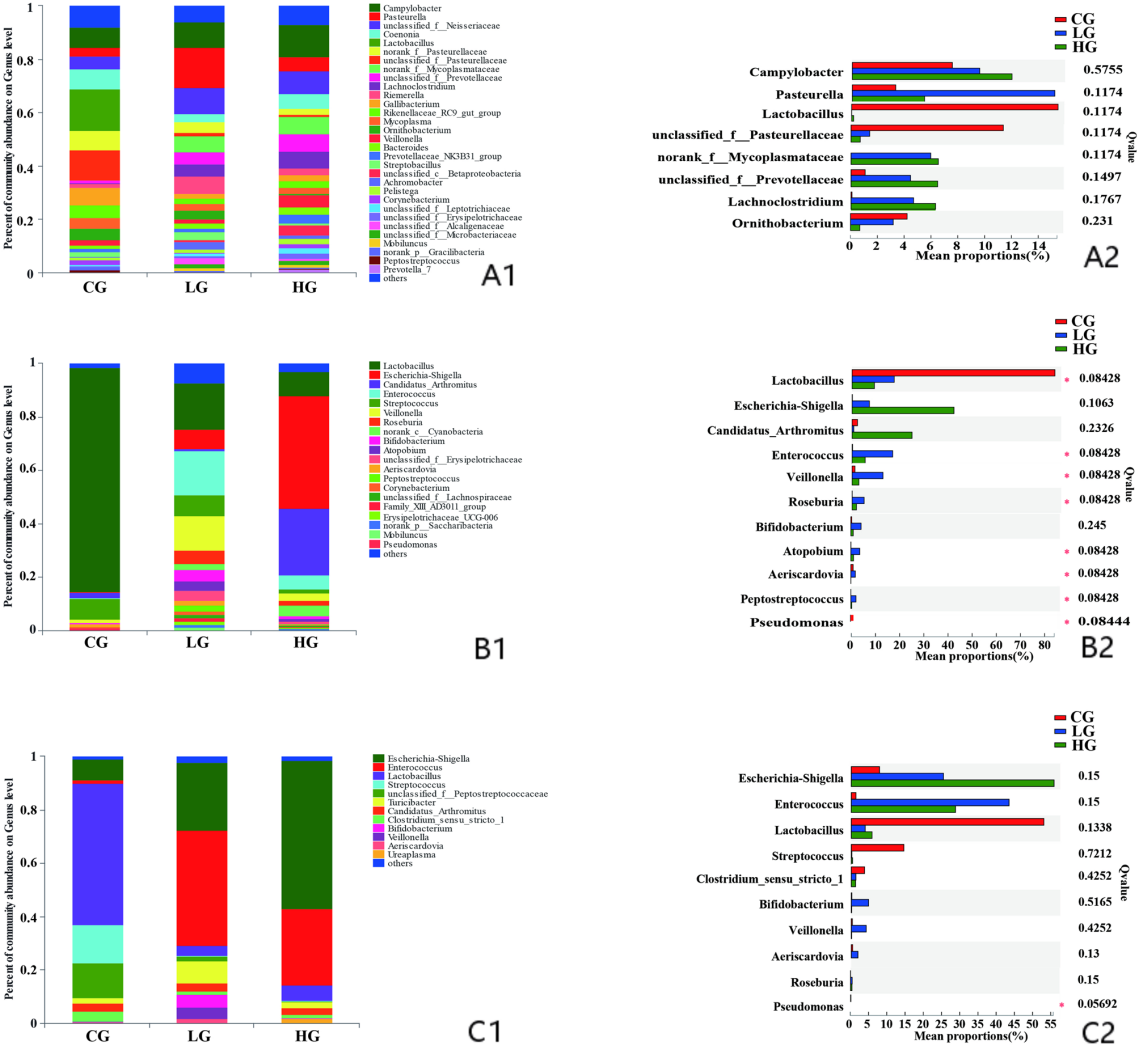
Changes in the relative abundances of the main bacterial communities (n = 18) on Genus level among the CG, LG and HG infection groups at the age of 21 days; (**A1**) community bar plot of crop samples; (**A2**) Kruskal–Wallis H test bar plot of crop samples; (**B1**) community bar plot of small intestinal samples; (**B2**) Kruskal–Wallis H test bar plot of small intestinal samples; (**C1**) community bar plot of rectal samples; (**C2**) Kruskal–Wallis H test bar plot of rectal samples.

At the age of 21 days, there were no significant changes in the microbiota composition in the crop except the abundance of *Saccharibacteria* and *Verrucomicrobia* (Supplemental Fig. 2A2). The abundance of *Firmicutes* decreased significantly in the small intestine of animals with trichomonosis, and the percentage of *Proteobacteria* increased in the birds of LG group and then changed continuously when birds were severely infected (Supplemental Fig. 2B2). The abundance of *Actinobacteria* increased in the small intestine of animals with trichomonosis, but then decreased when birds were severely infected (Supplemental Fig. 2B2). In rectum, the abundance of *Firmicutes*, *Bacteroidetes* and *Fusobacteria* decreased significantly with the severity of trichomonosis (Supplemental Fig. 2C2). The percentage of *Proteobacteria* increased with the severity of trichomonosis (Supplemental Fig. 2C2). At the genus level, *Lactobacillus* decreased dramatically in the small intestine (*Q* = 0.08428) of infected birds (Fig. 9B2). Although the abundance of *Lactobacillus* also decreased in the crop and rectum (Fig. 9A1,C1), but there was no significant difference according to the corrected *P*-values in this study (Fig. 9A2,C2). The abundance of *Escherichia-Shigella* increased gradually with the severity of infection in the small intestine and rectum of birds (Fig. 9B1,C1), however the differences were not significant (Fig. 9B2,C2). The genus of *Pseudomonas* was decreased by parasite infection in the small intestine (*Q* = 0.0844) and rectum (*Q* = 0.0569).

## Discussion

The endogenous microorganisms in the GI tract have significant impacts on animal health, mainly being involved in nutritional, metabolic, physiological and immunological processes^29–31^. The population density and composition of intestinal microbes constantly change as affected by various factors including diet^32^, age^33^, diseases and the location within the GI tract^34^. The population density of intestinal microbes increases along the GI tract in human^35,36^, and various studies showed that ceca-derived samples of broiler chickens had higher microbial diversity than those from small intestine and crop^37–40^. However, the data in this study was not consistent with those for human and chickens. Shannon index showed that the microbiota of the crop was much more diverse than that of the small intestine and rectum in pigeon. Besides, the crop had significantly different library compositions, while the composition of the small intestinal and rectal bacterial floras did not differ. This suggested that the bacterial community in crop was unique in nestling pigeons. We speculated that this effect was caused by ‘crop milk’, which transmits diverse microbes from parent pigeons to nestlings. Further investigations are necessary to understand the functional microbiota in the crop of pigeons so as to improve host health.

The microbiota in crop, small intestine and ceca develops along with age in poultry^32,33,40^. However, the microbial richness and diversity in crop and small intestine were not altered by age in our study. We therefore speculated that it was important for early colonization in pigeons, and the feeding method of mouth-to-mouth by the parents facilitated early microbiota colonization in crop and small intestine for young pigeons.

It has been reported that *Firmicutes*, *Bacteroidetes* and *Proteobacteria* are the dominant phyla in the GI tract of poultry^41–45^. In chickens, *Firmicutes* (~ 78%) and *Proteobacteria* (~ 16%) were the predominant phyla in the crop, whereas *Firmicutes* (~ 50%), *Bacteroidetes* (~ 29%) and *Actinobacteria* (~ 10%) were predominant in the caecum^46^. Our present study showed that the crop of pigeon has a higher percent of *Proteobacteria* and *Bacteroidetes,* and lower percent of *Firmicutes* compared with the hindguts. In the case of adult individuals of *Opisthocomus hoazin*, the content of the crop was dominated by higher proportions of *Bacteroidetes*, and lower proportions of *Firmicutes* and *Proteobacteria* compared with that of ceca^47^. This indicated that the differences of microbiota compositions between crop and hinguts have been observed in many kinds of birds, which may be caused by the host itself and various environmental factors including pH, nutrition and VFA concentration in each compartment of GI tract^48,49^.

The presence of the crop in the GI tract has some advantages, such as feed storage and moistening^50^ as well as inhibition of pathogens by colonization^51^. The crop as the first barrier to the colonization of the microbiota is particularly important in terms of integrity and homeostasis of the microbiome of further sections in the GI tract. The majority of the microbiome inhabiting the crop of broiler chickens^41^ and laying hens^52^ is bacteria assigned to *Lactobacillus* species^40,53–55^. In our present study, microbiota diversity and composition in the crop of pigeon squabs were very abundant. In addition to containing *Lactobacillus* (7.49% at 14 days; 15.4% at 21 days), the crop also contains a high proportion of *Veillonella, norank_f__Pasteurellaceae* and *Campylobacter*. This was not consistent with the previous studies on broilers, and this discrepancy may be explained by the different physicochemical conditions between pigeons and broilers. Species belonging to genus *Veillonella* could be important probiotics in pigeon ‘milk’^56^. Species belonging to genus *Veillonella* have been characterized as having inhibitory activity against the enteropathogenic bacterial species. In our present study, the increased abundance of *Veillonella* in the crop of nestling pigeons could be due to the direct seeding of the microbiota from parent pigeons. Besides, a previous study in broiler chickens suggested that the crop is also a source of *Campylobacter*, a potential pathogen^57^.

Moreover, there was a higher percentage of the *Lactobacillus* genus in the small intestine and rectum than in the crop of pigeons in this study. The percentages of *Lactobacillus* in the small intestine and rectum of 14-day-old pigeons were 81.02% and 67.03%, respectively. The proportions of *Lactobacillus* in the small intestine and rectum of 21-day-old pigeons were 83.94% and 52.87%, respectively. The abundance of *Lactobacillus* in the hindgut of nestling pigeons was significantly increased compared with that in the crop content. It has been showed that *Lactobacillus* species accounted for 67% of the total 16S rDNA sequences in the ileum of broiler chickens^35^. Similar studies focused on the small intestinal microflora, and illustrated that *Lactobacilli* was the abundant genus in this section of chickens^49,58^. However, Maki et al.^59^ found that the dominant microbiota was the family of *Lachnospiraceae*, *Peptostreptococcaceae* and *Enterococcaceae* in the jejunum and ileum of white leghorn layers at weeks 3 and 6 old, not *Lactobacillaceae* family or *Lactobacillus* genus. The reasons for inconsistent results may be that the composition of intestinal microbiota is influenced by many factors, such as diet, breed, the age of birds and the methods of sample processing^32,33^.

In pigeon production, the impacts of *T. gallinae* infection on the health of pigeons, especially pigeon squabs, have been reported a lot^7,8,21^. Although no obvious clinic symptoms were observed with infection by *Trichomonas* in pigeons from the LG and HG group, there was a large effect of *T. gallinae* on the microbiota diversity and composition in crop and small intestine of squabs at 14 and 21 days old. The digestive tract of pigeons is different from that of other poultry, for example, the cecum is almost degenerated, and we found that the greatest abundance of microbial species was mainly distributed in the crop, which was also the main site of *Trichomonas* infection. In present study, the infection significantly reduced the microbiota richness in crop of pigeons at 14 days old, and the percentage of *Lactobacillus* was reduced by at least 90% in infected pigeon squabs. Notably, there were more obvious differences in the small intestine than in the rectum and crop for pigeons at 21 days old during *Trichomonas* infection. With increasing degree of infection, *Firmicutes* abundances were decreased in the small intestine and rectum of pigeons at 21 days old, while the *Proteobacteria* phylum increased significantly, which were consistent with the report by Barash et al.^60^ Similarly, the abundance of *Lactobacillus* in the small intestine of infected pigeons was significantly decreased compared with that of the control pigeons at 21-day-old. It is well known that *Lactobacillus* is a kind of beneficial microbe that is especially critical for maintaining intestinal health by competing with pathogenic bacteria via adherence and replication^61^, producing anti-pathogenic compounds and regulating immunologic function^62–64^. The decrease of *Lactobacillus* abundance could lead to changing the intestinal environment dramatically, such as pH, and then the abundance of harmful bacteria appeared to increase. In present study, the genera abundances of *Enterococcus* and *Atopobium* were significantly increased in the small intestinal tract of *Trichomonas* infected birds, especially in LG group. It was suggested that *Enterococcus* species emerged as important pathogens in chicken, duck and pigeon due to their intrinsic or acquired resistance to a number of antibiotics^65–67^. *Atopobium vaginae* may be the marker for the bacterial vaginosis of woman^68^. Besides, *T. vaginalis* was reported to be associated with vaginal microbiota consisting of low proportion of *lactobacilli* and high proportions of *Mycoplasma, Parvimonas, Sneathia*, and *Atopobium*^69^.

Interestingly, the abundances of *Roseburia, Aeriscardovia* and *Peptostreptococcus* were also increased in the small intestine of LG infected pigeons, but they were decreased in those of HG group. The butyrate producing *Roseburia*^70^ were negatively associated with the diarrhea indices in mice, indicating *Roseburia* may have an effect of anti-diarrhea in GI tract^71^. The genus of *Aeriscardovia* belongs to the family *Bifidobacteriaceae*^72^, thus it may have potential probiotic functions. Wlodarska et al.^73^ reported that *Peptostreptococcus* species metabolized tryptophan to produce indoleacrylic acid, which promoted the function of intestinal epithelial barrier and alleviated inflammatory responses of immune cell. However, *Peptostreptococcus* species were also reported to be recovered in mixed infections involving the skin, respiratory tract, and gastrointestinal tract^74^. The roles of *Roseburia, Aeriscardovia* and *Peptostreptococcus* in the small intestine of piegeons infected with *T. gallinae* needs to be further studied.

In conclusion, the results indicated that crop had higher microbiota diversity than small intestine and rectum in pigeons, and *T. gallinae* infection decreased the microbiota richness in the crop of birds at 14 days old. The abundance of *Lactobacillus* genus was decreased, but *Enterococcus, Atopobium, Roseburia, Aeriscardovia* and *Peptostreptococcus* were increased in the small intestine of infected birds at 21 days old. This study provided fundamental knowledge of the bacterial flora in the digestive tract of late-maturing pigeon squabs, and the effects of *T. gallinae* infection on the diversity and composition of the microbiota in GI tract.

## Materials and methods

### Animals and treatment procedure

The study was carried out in accordance with the guidelines set by the Animal Care and Use Committee (permit number: SYXK-2017-0005) of the Institute of Animal Husbandry and Veterinary Medicine, Beijing Academy of Agriculture and Forestry Sciences (IAHVM-BAAFS), Beijing, China. The protocols were approved by the Animal Care and Use Committee of IAHVM-BAAFS.

### Birds, diets and treatments

Meat-type pigeon squabs (hybrid line) were sampled from a commercial pigeon farm (Miyun District, Beijing). Thirty-six squabs were involved in the study. Half of the birds were 14 days old, and the others were 21 days old. The squabs of the same age were separated into three groups (six squabs each), including healthy birds CG, LG and HG trichomonosis birds.

Diagnosing *T. gallinae* traditionally depends on direct microscopic observation of motile protozoa via wet mount preparation (i.e., immediate examination of glass slides). *T. gallinae* appear as elongated, oval shapes that move briskly. Samples from the crop of the birds were taken with a cotton swab to detect the presence of the protozoa in the upper digestive tract of squabs. All sampling swabs were diluted with 0.3 mL of physiological saline, then mounted on a slide and covered with an 18 mm square coverslip. Each sample was microscopically examined (400 × magnification). Positive samples were further examined to ascertain the severity of trichomonosis in birds. In the absence of clinical reference values for pigeons to date, we based by ourselves on the severity signs of *T. gallinae* infection. The method of determining the severity of disease was as follows: an average number of ten microscopic fields of each sample counted more than 20 was considered to represent the HG infection; an average counted less than 5 represented the LG infection.

According to the farming methods used by the pigeon industry, the parent pigeons were fed a formulated diet consisting of maize, peas, soybean meal and wheat. The feed, sand and water were provided ad libitum. Two squabs were raised by a pair of pigeons. Birds were housed in a room under a lighting cycle of 16 h light and 8 h darkness. The mean daily temperature was 22 ± 6 °C.

### Sample collection

A fresh crop swab was collected from each pigeon squab and stored in sample preservation buffer (TIANGEN Biotech CO., LTD, Beijing, China) at − 80 °C until analysis. Then, the squabs in each group were killed by cervical dislocation. The small intestine was defined as the segment from the duodenal loop to the ileocecal junction. The small intestine and rectum were tightly tied with string, and the contents were collected. The content samples were snap-frozen in sterile containers in liquid nitrogen and stored at − 80 °C for further analysis. A total of 54 samples, including crop swab, small intestinal and rectal contents, were collected from birds at 14 days and 21 days of age, respectively. All of the samples were used to analyze the microbiota composition and diversity.

### Microbiota diversity analysis

#### DNA extraction and PCR amplification

Total bacterial DNA was extracted from the collected crop swab and intestinal content samples using an E.Z.N.A. Stool DNA Kit (Omega Bio-tek, Norcross, GA, USA) according to the manufacturer’s protocol. The final DNA concentration and purity were determined by a NanoDrop 2000 UV–Vis spectrophotometer (Thermo Scientific, Wilmington, USA), and DNA quality was checked by 1% agarose gel electrophoresis. The V3-V4 hypervariable regions of the bacterial 16S rRNA gene were amplified with primers 338F (5′-ACTCCTACGGGAGGCAGCAG-3′) and 806R (5′-GGACTACHVGGGTWTCTAAT-3′)^23,24^, where barcode was an eight-base sequence unique to each sample. The PCR reactions were conducted (GeneAmp 9700, ABI, USA) using the following program: 3 min of denaturation at 95 °C, 27 cycles of 30 s at 95 °C, 30 s for annealing at 55 °C, and 45 s for elongation at 72 °C, and a final extension at 72 °C for 10 min. PCR reactions were performed in triplicate with 20 μL mixture containing 4 μL of 5 × FastPfu Buffer, 2 μL of 2.5 mM dNTPs, 0.8 μL of each primer (5 μM), 0.4 μL of FastPfu Polymerase, 0.2 μL of BSA, 10 ng of template DNA and double-distilled water^24,25^. The PCR products were extracted from a 2% agarose gel, further purified using an AxyPrep DNA Gel Extraction Kit (Axygen Biosciences, Union City, CA, USA) and quantified using QuantiFluor-ST (Promega, USA) according to the manufacturer’s protocol^25^.

#### Illumina MiSeq sequencing

Purified amplicons were pooled in equimolar amounts and paired-end sequenced (2 × 300) on an Illumina MiSeq platform (Illumina, San Diego, USA) according to the standard protocols^26^ by Majorbio Bio-Pharm Technology Co., Ltd. (Shanghai, China). The raw reads were deposited into the NCBI Sequence Read Archive (SRA) database (accession number: SRP200313).

#### Processing of sequencing data

Raw fastq files were quality filtered by Trimmomatic and merged by FLASH software (version 1.2, https://ccb.jhu.edu/software/FLASH/index.shtml)^24,25^. Operational taxonomic units (OTUs) were clustered with a 97% similarity cutoff using UPARSE (version 7.0, https://www.drive5.com/uparse/)^27^ analysis. The taxonomy of each 16S rRNA gene sequence was analyzed by the Ribosomal Database Project Classifier algorithm (version 11.1) against the Silva (SSU128) 16S rRNA database using 70% confidence threshold^28^.

### Statistical analyses

Alpha diversity (including Shannon, ACE, and Chao1 estimators) was calculated using Mothur. The ACE, Chao1, and Shannon indexes are presented for a similarity of 0.97 between reads. Differences between the estimators of the groups of days 14 and 21 were analyzed using Wilcoxon rank-sum test. Differences among the estimators of groups of crop, small intestine and rectum and those indexes of groups of CG, LG and HG infection of trichomonosis were tested, respectively, with Kruskal–Wallis *H* test. When significant effects were revealed, means were separated by Wilcoxon rank-sum test. Statistical significance was set at an adjusted *P*-value (*Q*-value) of < 0.1. And the difference was considered very significant if the *Q*-value was less than 0.05.

Distances between microbial communities in different samples were calculated using the unweighted_unifrac beta diversity metric via Mothur. Principal coordinates analysis (PCoA) was used to visualize the pairwise unweighted_unifrac distances among samples through R language (3.3.1, https://www.R-project.org).

The results of body weight were expressed as means ± standard deviations (SDs), and the data of bacterial abundances expressed as means. The microbiota compositions of the 14-day and 21-day group were analyzed with the Wilcoxon rank-sum test. The data from groups of CG, LG and HG trichomonosis and data from groups of crop, small intestine and rectum segments were compared, respectively, using the Kruskal–Wallis H test followed by Tukey’s post hoc multiple comparison test.

## Supplementary information

Supplementary figures.

## Data Availability

The sequences in this study have been deposited in the NCBI-SRA under BioProject PRJNA 546068, and the accession number is SRP 200313 for nestling pigeons.
