# Supplementary material for: Changes in the diversity and composition of gut microbiota in pigeon squabs infected with Trichomonas gallinae
Source: Sci Rep. 2020 Nov 17;10:19978. doi: 10.1038/s41598-020-76821-9 (PMC7673032; doi:10.1038/s41598-020-76821-9)
Supplement: Supplementary file 1 — Supplementary figures. [file 41598_2020_76821_MOESM1_ESM.docx]

**Changes in the diversity and composition of gut microbiota in pigeon squabs infected with *Trichomonas* *gallinae***

*Feng Ji^†^, Dongyan Zhang^†^, Yuxin Shao, Xiaohan Yu, Xiaoyong Liu, Dacong Shan^*^, Zheng Wang^*^*

*Institute of Animal Husbandry and Veterinary Medicine,* *Beijing Academy of Agriculture and Forestry Sciences,* *Beijing, China*

*^†^ These authors have contributed equally to this work.*

*^*^ Corresponding author. Tel.: +86 10 51 503 498; fax: +86 10 51 503 498. E-mail address: 1768301297@qq.com (DC. Shan).*

*^*^ Corresponding author. Tel.: +86 10 51 503 358; fax: +86 10 51 503 498. E-mail address: wz7324@163.com (Z. Wang).*

*Running title: Trichomonas and pigeon gut microbiota*

**Supplemental Figure legends**

Supplemental Figure 1. Changes in the relative abundances of the main bacterial communities (n=12) on Phylum level among the GI segments of control squabs; (A1) community bar plot at 14 days; (A2) Kruskal-Wallis H test bar plot at 14 days; (B1) community bar plot at 21 days; (B2) Kruskal-Wallis H test bar plot at 21 days.

Supplemental Figure 2. Changes in the relative abundances of the main bacterial communities (n=18) on Pylum level among the CG, LG and HG infection groups at the age of 21 days; (A1) community bar plot of crop samples; (A2) Kruskal-Wallis H test bar plot of crop samples; (B1) community bar plot of small intestinal samples; (B2) Kruskal-Wallis H test bar plot of small intestinal samples; (C1) community bar plot of rectal samples; (C2) Kruskal-Wallis H test bar plot of rectal samples.


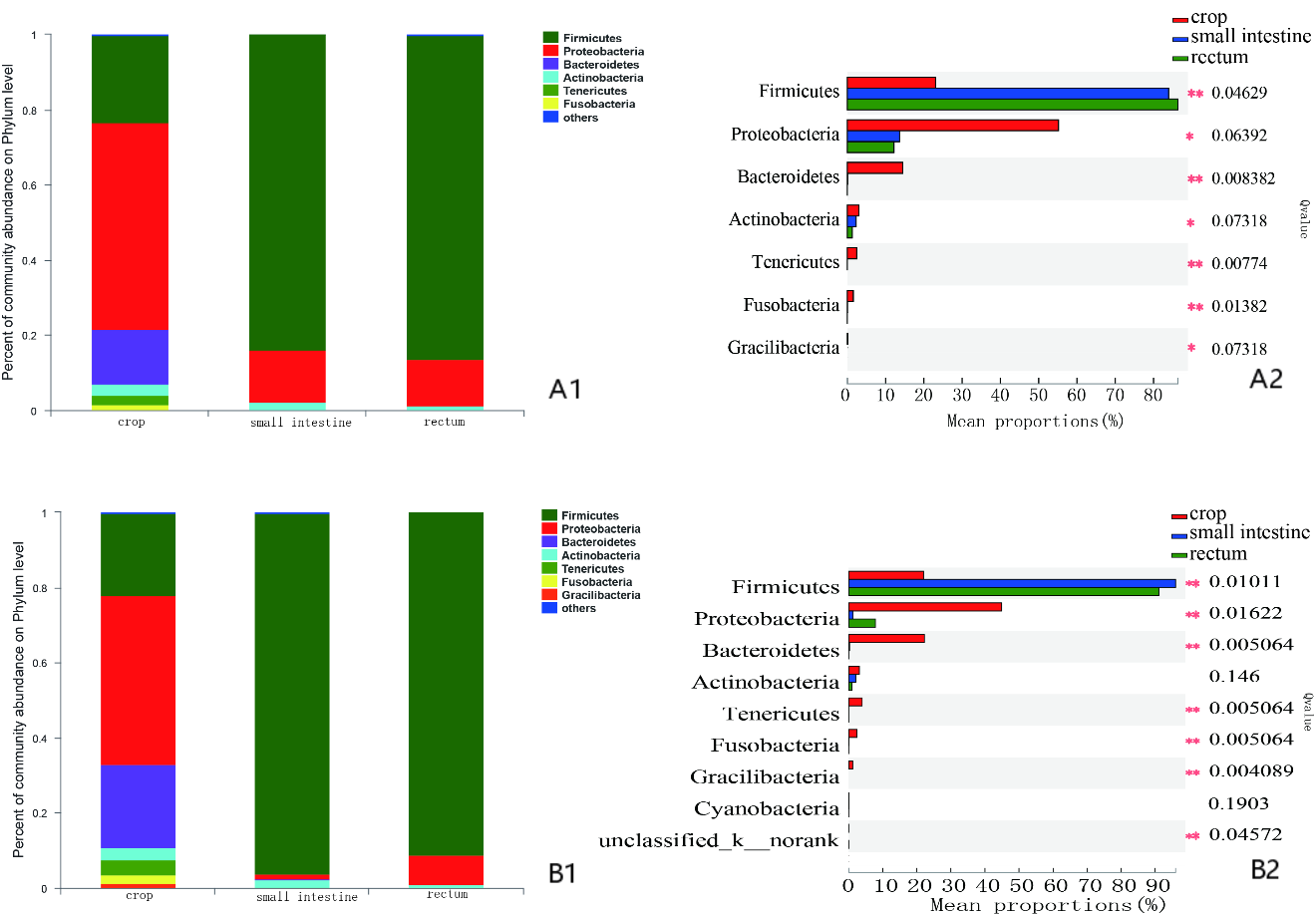


Supplemental Figure 1 Changes in the relative abundances of the main bacterial communities (n=12) on Phylum level among the GI segments of control squabs; (A1) community bar plot at 14 days; (A2) Kruskal-Wallis H test bar plot at 14 days; (B1) community bar plot at 21 days; (B2) Kruskal-Wallis H test bar plot at 21 days.


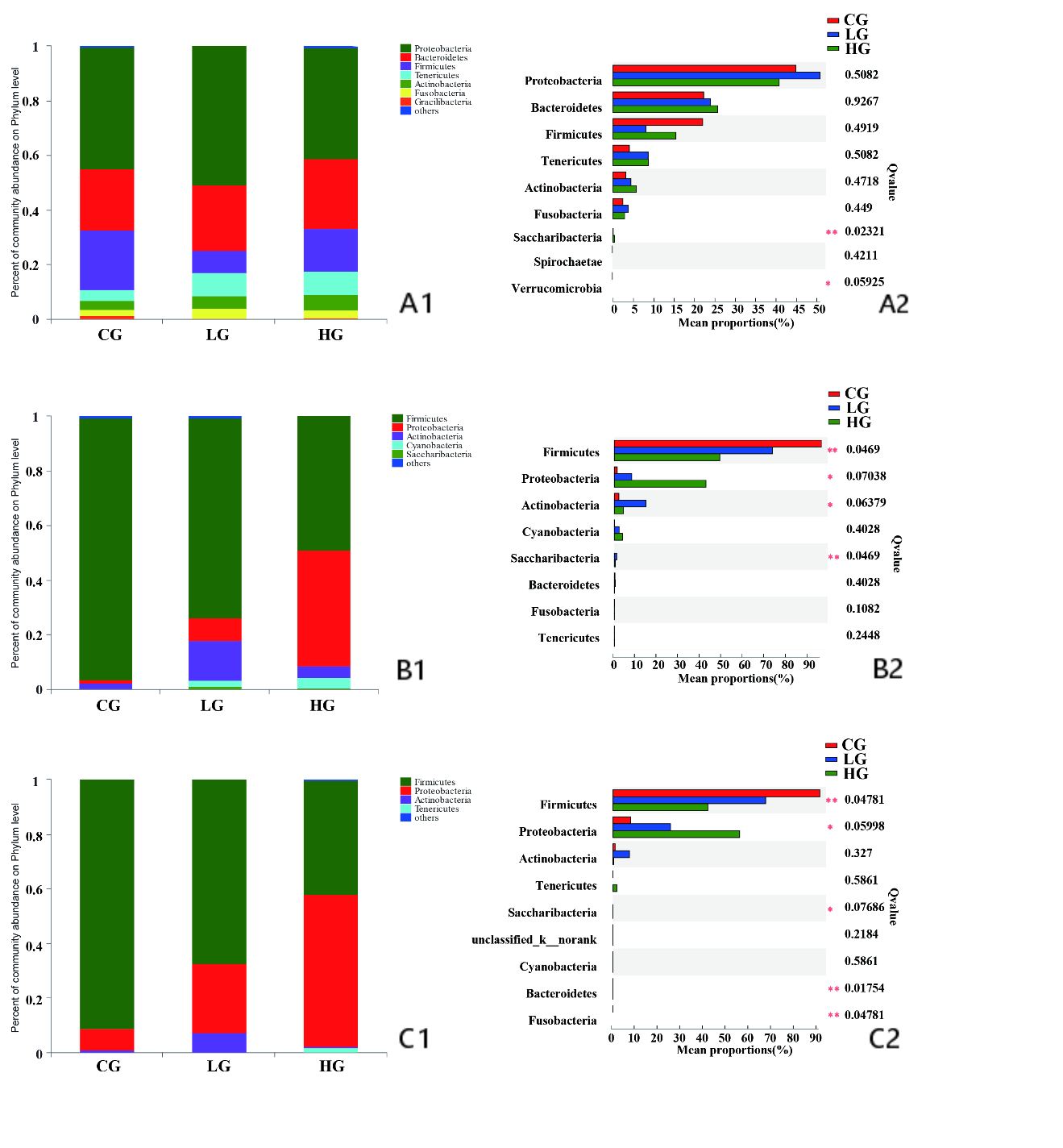


Supplemental Figure 2. Changes in the relative abundances of the main bacterial communities (n=18) on Pylum level among the CG, LG and HG infection groups at the age of 21 days; (A1) community bar plot of crop samples; (A2) Kruskal-Wallis H test bar plot of crop samples; (B1) community bar plot of small intestinal samples; (B2) Kruskal-Wallis H test bar plot of small intestinal samples; (C1) community bar plot of rectal samples; (C2) Kruskal-Wallis H test bar plot of rectal samples.
